# Supplementary material for: Diffantom: Whole-Brain Diffusion MRI Phantoms Derived from Real Datasets of the Human Connectome Project
Source: Front Neuroinform. 2016 Feb 5;10:4. doi: 10.3389/fninf.2016.00004 (PMC4742542; doi:10.3389/fninf.2016.00004)
Supplement: Supplementary file 3 [file Appendix.pdf]

Supplementary Material to: Diffantom

Oscar Esteban*, Emmanuel Caruyer, Alessandro Daducci, Meritxell Bach-Cuadra,María-J. Ledesma-Carbayo and Andres Santos

*Correspondence: Oscar Esteban (phd@oscaresteban.es)

APPENDIX

Let $\{T'_j\}$ be the set of original fractions maps obtained with `act_anat_prepare_fsl`, a tool in *MRTrix* that combines FAST (FMRIB's Automated Segmentation Tool, Zhang et al., 2001) and FIRST (FMRIB's Integrated Registration and Segmentation Tool, Patenaude et al., 2011) to generate the macrostructural 5TT map. FA denotes the fractional anisotropy (FA) map obtained from the original diffusion MRI (dMRI) data: the local fiber orientation maps $\{\mathbf{V}_i\}$ with their estimated volume fractions $\{F'_i\}$ calculated with BEDPOSTX (Bayesian Estimation of Diffusion Parameters Obtained using Sampling Techniques modelling crossing –X– fibres, Jbabdi et al., 2012). The final $\{T_j\}$ maps of isotropic fractions are computed as follows:

$$
\begin{aligned}
T_1 &= (1.0 - f_{cgm}) \cdot T'_1 \\
T_2 &= (1.0 - f_{dgm}) \cdot T'_2 \\
T_3 &= (1.0 - f_{wm}) \cdot T'_3 \\
T_4 &= T'_4 \\
T_5 &= 0.0
\end{aligned}
$$

where $f_{\{cgm,dgm,wm\}}$ are the fractions of restricted diffusion for each tissue. Sepehrband et al. (2015) found out that the fiber fraction ranges across the corpus callosum from the 70±8% in its body to an upper bound of 80±11% in the splenium. Therefore, we choose $f_{wm} = 80\%$ as default fraction of restricted diffusion in the white matter (WM). To our knowledge, restricted diffusion fractions have been studied only for WM. Therefore, we set $f_{cgm} = 25\%$ and $f_{dgm} = 50\%$ as they yield plausible FA and anisotropic diffusion coefficient (ADC) maps, assessed visually. The final $\{F_i\}$ maps are computed as follows:

$$
\begin{aligned}
F_1 &= f_{wm} \cdot T_2 \cdot \text{FA} + w_{f1}(f_{cgm} \cdot T_1 + f_{dgm} \cdot T_2) \\
F_2 &= f_{wm} \cdot T_2 - (F_1 + F_3) + w_{f2}(f_{cgm} \cdot T_1 + f_{dgm} \cdot T_2) \\
F_3 &= f_{wm} \cdot F'_3 + w_{f3}(f_{cgm} \cdot T_1 + f_{dgm} \cdot T_2)
\end{aligned}
$$

where $w_{\{f1,f2,f3\}}$ are the contributions of the gray matter (GM) compartments to each fiber population. By default: $w_{f1} = 48\%$, $w_{f2} = 37\%$, $w_{f3} = 15\%$. Finally, the resulting maps are normalized to fulfill $\sum_j T_j + \sum_i F_i = 1.0$.

REFERENCES

Jbabdi, S., Sotiropoulos, S. N., Savio, A. M., Graa, M., and Behrens, T. E. J. (2012). Model-based analysis of multishell diffusion MR data for tractography: How to get over fitting problems. *Magn Reson Med* 68, 1846–1855. doi:10.1002/mrm.24204

Patenaude, B., Smith, S. M., Kennedy, D. N., and Jenkinson, M. (2011). A Bayesian model of shape and appearance for subcortical brain segmentation. *NeuroImage* 56, 907–922. doi:10.1016/j.neuroimage.2011.02.046

Sepehrband, F., Clark, K. A., Ullmann, J. F., Kurniawan, N. D., Leanage, G., Reutens, D. C., et al. (2015). Brain tissue compartment density estimated using diffusion-weighted MRI yields tissue parameters consistent with histology. *Hum. Brain Mapp.* 36, 3687–3702. doi:10.1002/hbm.22872

Zhang, Y., Brady, M., and Smith, S. (2001). Segmentation of brain MR images through a hidden Markov random field model and the expectation-maximization algorithm. *IEEE Trans Med Imag* 20, 45–57. doi:10.1109/42.906424
